# Decoding cellular population dynamics through mechanistic modelling and statistical data analysis

**DOI:** 10.1038/s41540-026-00751-x

**Published:** 2026-05-25

**Authors:** Nissrin Alachkar, Nicholas Kwasi-Do Ohene Opoku, Nicholas A. M. Monk, Kevin Thurley

**Affiliations:** 1 Biomathematics Division, Institute of Experimental Oncology, University Hospital Bonn, Bonn, Germany; 2 African Institute of Mathematical Sciences, Accra, Ghana; 3 School of Mathematical and Physical Sciences, University of Sheffield, Sheffield, UK; 4 Bonn Center for Mathematical Life Sciences, University Bonn, Bonn, Germany

**Keywords:** Cell biology, Computational biology and bioinformatics, Mathematics and computing, Systems biology

## Abstract

Cell-cell communication underlies key processes in development, immunity, and disease, yet capturing its mechanistic complexity remains challenging. While advances in single-cell omics have revealed new insights into cell-type diversity, mathematical modelling has become essential for deriving mechanistic understanding of their communication networks. Here, we overview established modelling approaches and highlight the need for frameworks that move beyond steady-state assumptions and single-step processes, better reflecting the nature of cell–cell communication.

## Introduction

Life in multicellular organisms depends on the precise orchestration of cell-cell communication. Key processes in development, physiological functions, immune responses, and disease progression are coordinated through such intricate interactions^1^. Communication between cells occurs through several mechanisms, including direct cell–cell contact, the secretion and uptake of signalling molecules such as cytokines and chemokines, and the release of extracellular vesicles. In turn, the spatial and temporal dynamics of these mechanisms shape downstream cellular responses^2^.

High-throughput analysis of individual cells has become feasible through technological advances including multicolour flow cytometry, single-cell transcriptomics, and spatial profiling using state-of-the-art multiplexed imaging technologies for both proteomics and transcriptomics^3^. Each provides complementary types of data that give insights into cell–cell communication pathways. Advanced computational tools and machine-learning methods based on statistical and bioinformatics principles are now widely employed for processing, analysing, visualising, and extracting quantitative patterns and features of these high-dimensional datasets^4–9^. In practice, a wide range of methodologies is available, depending on the analytical task. For established workflows, including dimensionality reduction, clustering, trajectory inference, and gene regulatory network inference, we refer the reader to comprehensive reviews^2,3,10,11^. Moving beyond static cell-state descriptions, quantitative frameworks inspired by Waddington’s epigenetic landscape^12^ provide a principled way to link experimentally observed cell states to the dynamical processes governing transitions between them. For example, pseudodynamics models infer population-level dynamics on differentiation landscapes from time-resolved single-cell RNA sequencing data^13^, enabling quantification of proliferation and apoptosis rates during T-cell maturation and identification of key developmental checkpoints. Other methods map pseudotime-ordered cells onto real temporal and spatial scales^14–16^. Approaches based on optimal transport theory have been developed to infer trajectories of gene-expression programmes rather than trajectories of cells^17,18^. Furthermore, integrative computational frameworks have been developed to jointly analyse heterogeneous datasets across multiple resolutions, including population-level, single-cell, temporal, and spatial measurements. These approaches have been successfully applied to dissect regulatory mechanisms underlying immune control in diverse disease contexts^9,19–24^. Together, data-driven techniques serve as an essential bridge between high-dimensional measurements that are rich in information and their biological interpretation.

Nonetheless, dissecting complex communication networks comprehensively requires mapping not only the identities and states of interacting cell populations, but also their kinetics and feedback topologies within spatially heterogeneous and dynamic microenvironments^2,25–29^. Mechanistic mathematical modelling offers a complementary means to derive a quantitative understanding of complex dynamical processes, and to generate mechanism-based predictions aiding experimental design and optimised therapeutic strategies^30–32^. Such modelling approaches span a broad spectrum of methodological frameworks. They include ordinary differential equation (ODE) systems describing temporal dynamics at the population level, and stochastic models capturing cell-to-cell variability and intrinsic noise, as well as spatiotemporal frameworks such as reaction–diffusion models or the Cellular Potts model (CPM)^33–37^. Ultimately, the choice of the modelling approach depends on the specific biological question asked, the assumptions made when constructing the model, and the level of mechanistic detail required to answer the research question.

Here, we discuss how the integration of these modelling techniques with experimental measurements is advancing our understanding of cell–cell communication in health and disease, with illustrative examples from infectious diseases, cancer immunology, and developmental biology. In addition, we outline the growing need for advanced mathematical and computational modelling techniques, due to the increasing complexity of experimental datasets and accumulating evidence that biological processes operate far from steady state. Finally, we provide our perspective on the classical data–model cycle and offer an outlook on this rapidly evolving field.

## Why model? - Mathematical tools and workflows to decipher tissue-scale dynamical mechanisms

**Fig. 1:**
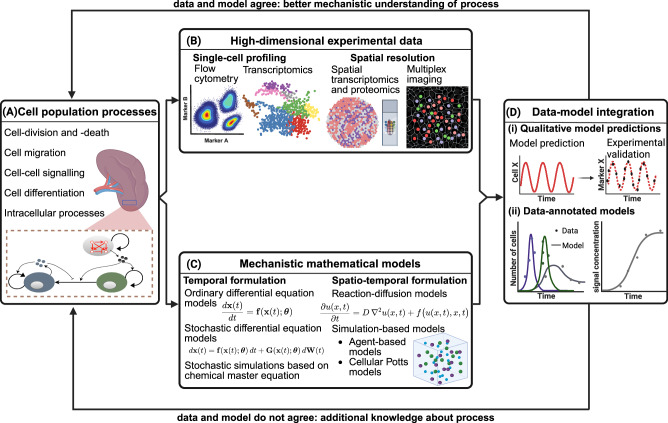
Conceptual workflow of data-model integration in cell-population dynamics. **A** Cell-cell communication arises from intertwined processes operating across multiple scales, from tissue-level organisation to inter- and intracellular signalling. **B** High-throughput experimental data enable profiling of cellular populations, including their transcriptomic and proteomic states at single-cell resolution across time and space. The analysis of high-dimensional datasets requires dimensionality reduction techniques. **C** The study of complex processes calls for simplified, tractable models. Mechanistic mathematical models provide a framework enabling the identification of fundamental regulatory mechanisms and quantitative interpretation through temporal and spatiotemporal formulations. **D** Although data-driven analyses and model-based analyses can be performed independently to yield insights into cell–cell communication, their integration enables a comprehensive and validated mechanistic understanding of the underlying processes. Created in BioRender. Alachkar, N. (2026) https://BioRender.com/6nyhenw.

Cell–cell communication networks are inherently complex systems, shaped by inter- and intracellular processes that are in turn governed by history- and space-dependent responses, feedback-driven dynamics, and cell-intrinsic noise. In addition, the informational richness of high-dimensional and spatially resolved measurements, together with their snapshot-based nature, implies that biological insight cannot be extracted without rigorous frameworks capable of reducing dimensionality and revealing underlying mechanisms. Studying such systems therefore requires navigating both the spatiotemporal complexity of the underlying biology and the difficulty of inferring insight from immense datasets. Models provide a systematic methodology to address this challenge. Abstracting a biological system into a simplified representation allows integration of existing knowledge into a coherent and interpretable structure that can be systematically interrogated and refined. While this abstraction already offers qualitative insight, formalising components and interactions through mathematical equations enables quantitative analysis with a wide range of mathematical and computational techniques. Figure 1 illustrates integration of experimental and mathematical approaches to study biological systems, including cell-population processes. While experiments and modelling can be considered and designed simultaneously, the workflow can begin from either data-driven analysis or mechanistic modelling, representing typical entry points in current practice, and converges through their integration. As part of this iterative workflow, agreement between model and data leads to improved mechanistic understanding, whereas disagreement drives model refinement and the generation of new biological hypotheses.

Quantitative analysis of a mathematical model using concepts from dynamical systems theory, including equilibrium and stability, phase-plane trajectories, and sensitivity and bifurcation analysis, provides a deeper understanding of system behaviour and allows for the identification of critical transitions^38^. This approach has been utilised to characterise so-called network motifs, defined as recurring and statistically overrepresented patterns of interconnections within complex molecular and cellular networks^39,40^. Examples of network motifs include feedforward loops, feedback loops, and mutual inhibition circuits, which serve as fundamental building blocks of biological communication networks^41,42^. The repeated occurrence of network motifs across different biochemical pathways, and even species, suggests that they represent functionally important regulatory sub-networks implementing specific dynamical behaviours^43^. Further, they have been proposed to facilitate efficient evolutionary optimization by providing reusable functional modules that can be deployed across various biological contexts^44^. In the context of immune-cell populations, systematic analysis of such network motifs has revealed core circuit-design features for controlled immune-cell responses, including robustly tuned population growth and stable perturbation-resilient dynamics^45–49^. Further, modelling studies of one of the central regulators of inflammatory responses, the nuclear factor kappa-light-chain-enhancer of activated B-cells (NF-κB) signalling pathway, have unravelled multiple feedback loops governing its dynamics^50,51^. In particular, negative feedback mediated by inhibitor of NF-κB has been shown to drive the characteristic oscillatory behaviour of the NF-κB system^52–55^.

Experimental data yield rich information about cell–cell communication, while mathematical models offer mechanistic explanations of how these systems evolve and interact across time and space. Integrating data with models is therefore essential to maximise the information gained from both. Model-driven predictions from simulations and theoretical analyses can guide the design of targeted experiments to investigate qualitative behaviour, such as oscillations or abrupt state-changes occurring in certain parameter regimes. Conversely, constraining models with experimental data increases confidence that they capture the key elements governing the underlying biological mechanism. A range of mathematical techniques is available to perform the task of model fitting^56,57^. These include widely implemented optimisation methods, such as least-squares fitting and genetic algorithms, which seek the set of parameters that minimises the discrepancy between model outputs and experimental data. Another approach is the maximum likelihood estimation method, a method grounded in statistical theory that allows for testing the identifiability of model parameters^58,59^. When designing experiments, it is important to note that high-resolution data collected across multiple time points and conditions is especially valuable for parameterising models and distinguishing between competing hypotheses. To ensure robust inference of model parameters and avoid overfitting, the number of data points should meet or exceed the number of fitted parameters, and sampling should be sufficiently dense, particularly during periods of rapid change in the system’s state. Overall, increasing interdisciplinary efforts have made it clear that combining theory and experiments already in the design of a research project is a big advantage for deriving mechanistic and quantitative insights into cell-cell communication processes. We illustrate the importance of theory-data integration in the following examples.

In immunity and infectious diseases, mechanistic models integrated with or driven by experimental data have elucidated key regulatory principles shaping the complex dynamics of cell-cell communication by quantitatively describing interactions between pathogens, host immune responses, and intracellular signalling within immune cells. For instance, multi-scale models of SARS-CoV-2 infection have been developed to capture the interactions between viral dynamics and immune responses at both inter- and intracellular levels^60–62^. Model analyses revealed immune-regulatory mechanisms underlying disease outcomes^60^, and key modulators of patient-to-patient heterogeneity^61^. These insights enabled further in silico investigation of therapeutic efficacy and effects, leading to the identification of optimised treatment strategies tailored to disease severity and amenable to clinical testing^62^. In a similar fashion, recent studies have quantified the nonlinear regulatory mechanisms governing immune responses to influenza viruses, including the spatiotemporal aspects of virus detection and control^63–65^. In particular, infection spread patterns of the influenza A virus within the human respiratory tract have been predicted by constructing and analysing a spatiotemporal model that incorporates diffusion and advection of viral infectious particles, with predictions validated by real-time imaging data from infected mice models^66^. Complementarily, cytotoxic T lymphocyte (CTL)–mediated killing of virus-infected cells has been studied using three-dimensional cellular automaton models that capture cell movement and interactions within the spleen tissue^67^. Model analyses revealed that CTL killing efficacy is primarily limited by the time required to kill an infected cell. While informed by microscopy data, these models also provide a framework for designing future experiments focused on model-predicted mechanisms underlying CTL-mediated killing. Furthermore, a Cellular Potts model demonstrated that chemokine-driven migration of T cells towards antigen-presenting dendritic cells, an essential step for T-cell activation, enhances the efficiency of interactions between these specific cell types in the presence of numerous competing cells, thereby promoting the detection of rare antigens^68^.

At the intracellular level, regulatory mechanisms involving specific networks of transcription factors governing transcriptomic responses in fibroblasts and macrophages were uncovered through data-driven approaches^69^ and iterative modelling of gene regulatory networks embedded within an ODE framework, further refined using transcriptomic datasets^70^. Moreover, the regulatory control of the pro-inflammatory cytokine tumour necrosis factor (TNF), secreted by macrophages in response to bacterial infection, was investigated by modelling TNF propagation in tissues combined with time-lapse microscopy and single-molecule RNA fluorescence in situ hybridization data^71^. The study showed that competitive uptake of TNF by the surrounding environment effectively constrains signal diffusion, thereby preventing chronic inflammation. In addition, by combining stochastic modelling with single-cell transcriptomics, studies have shown that transcriptional variability of TNF and other cytokines in the Toll-like receptor signalling pathway is closely linked to gene regulatory complexity^72,73^. Genes exhibiting higher variability required more detailed gene expression models, whereas genes with lower variability were well captured by the telegrapher’s model, a simpler formulation of gene regulation.

Cancer immunology has similarly been advanced through the integration of mathematical modelling for the study of tumour-immune interactions across inter- and intracellular levels^74–78^. For example, a simplified model of tumour interactions with helper and cytotoxic T cells has shown that tumour elimination, equilibrium, or escape depend strongly on the infiltration rates, with combination therapies potentially acting synergistically to control tumour growth^79^. In addition, analyses of an agent-based model of macrophages and tumour cells using a newly developed spatial statistic revealed that macrophage-tumour interactions are affected by their spatial organisation and macrophage phenotypic heterogeneity^80^. A comprehensive ODE model considering interactions between multiple immune cell types, key cytokines and tumour cells revealed that tumour antigenicity critically shapes efficacy of the immune response^81^. Building on this model, subsequent work incorporated therapeutic interventions, including the role of IL-2 in adoptive cell transfer therapy and the effects of combining immunotherapy and chemotherapy^82^. Other studies have applied similar frameworks to investigate cancer–immune dynamics under various therapeutic conditions, demonstrating the power in integrating high-dimensional single-cell data analysis with data-informed mechanistic models to advance precision immunotherapy^27,83,84^.

Collectively, modelling approaches together with data provide tractable frameworks for systematically probing system dynamics and uncovering emergent biological phenomena^30^. Moving forward, integrating temporal, spatial, and stochastic aspects of communication within a unified framework will require innovative multiscale and hybrid modelling approaches, supported by rigorous parameter inference and validation using high-throughput longitudinal experimental data.

## Beyond the equilibrium assumption—long transients, delays and spatial patterns

Mathematical models of interactive cell–population dynamics have successfully generated biological explanations, hypotheses, and predictions. Nevertheless, an accurate description of the multi-scale processes involved imposes considerable challenges for model annotation, simulation and interpretation of results. Notably, the involved processes range from gene expression to intracellular signal transduction to cell-cell communication, proliferation, movement, death, and differentiation. In order to still apply well-established mathematical tools, many of the examples discussed above made use of the assumptions of well-separated timescales, well-mixed conditions of extracellular ligands, or single-step reactions replacing intracellular dynamics, which are not always justified in immune-cell populations.

In particular, in dynamic tissue environments, biological systems operate in regimes where timescales are tightly coupled; moreover, complex intracellular signalling networks often give rise to non-Markovian behaviour, manifesting as delays and even multi-modal waiting-time distributions^85–89^. To incorporate the effects of intracellular processes in studies of intercellular network dynamics, we developed a response-time modelling framework^49,89,90^ in which cell-state changes are represented as input–output relationships described by non-exponential probability distributions for the next-reaction time. These distributions can be derived directly from experimental time-course data, as from gene-expression or flow-cytometry readouts. For example, in our analysis of helper T-cell transcriptomics, we found that gene expression kinetics are often well captured by gamma distributions^49^, which can be incorporated into an ODE framework describing cellular dynamics using the linear-chain trick. Specifically, we investigated cell-fate decisions, proliferation, and intercellular communication among T-cell populations during acute and chronic LCMV infection by developing a mathematical model based on measured distributions as input data. This approach enables a mechanistic yet computationally tractable link between intracellular events and population-level behaviour. Complementary to such distribution-based modelling approaches, the recently developed density physics-informed neural networks method infers transduction-time distributions directly from single-cell response trajectories, revealing key features of the signalling pathway, including speed, structure, and how multi-timescale signalling architectures shape cell-to-cell heterogeneity in signal transduction^91^.

Furthermore, recent findings indicate that in the immune system, long transient behaviours often emerge^92,93^. As immune cells respond to fluctuating cytokines, antigen levels, and tissue cues, their interactions evolve within intrinsically time-dependent environments. This has prompted increasing interest in modelling approaches that explicitly incorporate for time-dependency in environmental cues^94^. Studies of cell polarisation illustrate this shift; cells exposed to dynamic chemoattractant fields exhibit transient memory and adaptive responses that extend beyond equilibrium-based descriptions^95–97^. Traditional frameworks such as local excitation–global inhibition^98^, Turing-like^99^, and mass-conserved reaction–diffusion models, including wave-pinning formulations^100,101^, have been instrumental in understanding spatial asymmetry and feedback mechanisms; however, they do not capture these long transient behaviours beyond the signal duration. A recent study on modelling cell polarity response offers a modelling framework that accounts for the long transient dynamics through studying the non-asymptotic dynamics of the system’s state trajectories in a non-autonomous treatment of the system^96^. In general, studying non-asymptotic transients reveals how systems poised near criticality can integrate memory, flexibility, and robustness, enabling cells to maintain functionality in dynamically changing environments.

Further, in many cell-signalling systems, pulsatile or oscillatory signals play an important role. In addition to the NF-κB system referred to earlier, oscillatory signalling is central to spatiotemporal coordination in the development of embryonic tissues such as the somites and the nervous system^102^. Combined modelling and experimental studies have revealed the critical influence of both intracellular and intercellular time delays and signalling stochasticity in these processes^103,104^. A theoretical study of how ligand-receptor systems and their associated signalling motifs and pathways respond to pulsatile inputs again reveals the potential importance of transient responses, with distinct frequency responses in transient and long-term regimes^105^. For instance, intercellular signalling in developing embryos typically operates in tissues in which cell division and cell movement rearrangements occur on timescales comparable to those of signalling responses, with tight coupling between all three processes^106,107^. The integration of these processes results in “microheterogeneity” – small variations in the signalling environment of cells^108^. Tracking and modelling cell signalling in such tissue contexts presents significant experimental and theoretical challenges, and recent progress highlights the opportunities presented by approaches that integrate advanced data analysis and modelling. Advanced fluorescence microscopy allows the visualisation of the movement of, and response to, secreted signalling molecules in complex tissues, providing detailed information on ligand distributions^109^.

In dynamic tissues, keeping track of intercellular signalling requires knowledge of how cell-neighbourhood relationships change over time due to cell divisions and cell movement, particularly when short-range signalling is involved. The use of synthetic signalling systems now provides multiple tools that allow the dynamic identification of neighbourhood relations in complex tissues^110^. These tools have recently been integrated into a computational toolkit that provides a detailed four-dimensional view of the signalling underlying the patterned differentiation of neuromesodermal progenitors in mouse embryos^111^. Importantly, the dimensionality reduction achieved by going from complex three-dimensional image data to segmentation and cell-neighbourhood data allows rational comparison between different embryos and with equivalent processes in cell culture systems and embryos of other species. Indeed, several studies have highlighted the importance of the interplay of cell-neighbourhood dynamics and signalling timescales on the tissue-level patterning of signalling outcomes in development. Examples include the importance of cell mixing in maintaining multicellular synchrony in the oscillatory signalling system operating in the presomitic mesoderm^112^ and the regulation of neuromesodermal precursor differentiation by the movement-mediated temporal profile of morphogen exposure^113^. Hence, the combination of new imaging and cell-tracking pipelines, single-cell expression profiling, and modelling provide exciting new avenues for exploring the generation of patterned cell fate through the interaction of cell movements and cell signalling^114^.

Simulations have shown that even in situations of randomly localised cells and quasi-steady-state conditions due to high diffusivity of ligands, cytokine gradients spanning several orders of magnitude can emerge^115^, while experimental findings have revealed spatially inhomogeneous signal propagation in lymphoid tissue^116,117^. That highlights the need for mathematical modelling approaches accounting for the full complexity of cell-population dynamics in tissue, while still being comparable to experimental data and accessible to systematic model analysis. Recent efforts have begun to model and quantify cytokine gradient formation^116,118,119^. For instance, we developed a three-dimensional spatiotemporal simulation framework based on the finite-element method, to investigate regulatory mechanisms and the impact of specific tissue architectures on the emerging spatial inhomogeneities of cytokine signalling^118^. As evidence continues to accumulate that cytokine diffusion, tissue heterogeneity, and spatial organisation fundamentally shape intercellular communication, and as rapid technological advances enable the generation and quantification of high-dimensional spatial datasets^9,120–122^, there is now a clear opportunity and need for a stronger focus on spatiotemporal modelling.

Overall, alongside technological advances in the acquisition, processing and statistically analysing tissue-scale high-content data, as well as the development of data-driven machine-learning and neural-network based approaches, considerable progress has been made on the side of mathematical tools and workflows. The examples discussed here illustrate the challenges in achieving a quantitative understanding of complex processes such as immune-cell-population dynamics in vivo. A key starting point is an adequate mathematical ‘language’ that takes problem-specific constraints into account and is formulated in terms of measurable quantities.

## Revising the classical data-model cycle

Which should come first, the data or the (mathematical) model? In practice, significant progress emerges not from prioritising one over the other, but from recognising that both are inseparable tools, two faces of the same coin, working in tandem to refine hypotheses and guide discovery. This integrative view complements the conventional data–model cycle, or as described in other work the forward and reverse modelling^123^, by reframing it as an iterative, co-evolving process where data inform models and models continuously shape experimental design.

Integrative approaches that leverage analysis of high-dimensional data to infer mechanistic insight, have already advanced our understanding of complex regulatory dynamics in various biological systems^70,73,124–128^. In particular, data-based modelling leads to uncovering regulatory principles that would otherwise remain overlooked or take substantially longer to identify, thereby accelerating mechanistic discovery. Other recent studies exemplify this synergy by combining statistical analysis of single-cell datasets with concepts from dynamical systems theory to infer the regulatory dynamics underlying cellular decision-making processes^129,130^. In particular, integrating statistical inference with catastrophe theory and approximate Bayesian computation has enabled the construction of quantitative dynamical landscapes that predict cell-fate decisions under distinct signalling conditions^131^. Moreover, Waddington landscape–based frameworks have been extended to incorporate explicit coupled PDE-ODE dynamical systems that capture cell–cell communication, thereby linking single-cell data with mechanistic descriptions of collective cellular dynamics^132^.

Achieving this iterative model–data integration requires close collaboration between experimentalists and theoreticians, or even training researchers to navigate both domains. Essentially, emerging datasets of increasing complexity are of limited value without suitable models and tools capable of capturing the underlying dynamics and mechanisms. In Box 1, we propose practical guidelines that can help structure such collaborations and guide productive interaction between the two communities.

When designing a model of a biological process, careful consideration must be given to its logical structure, which should be grounded in explicit assumptions derived from existing biological knowledge^123,133^. The assumptions made and the level of biological detail incorporated are ultimately guided by the specific research question and hypotheses, which in turn dictate the structural complexity of the model. Importantly, omitting certain biological details does not necessarily undermine a model’s validity; an appropriately simplified model can still capture the essential system dynamics while offering greater interpretability and analytical tractability^134^. In fact, beginning with the simplest possible model and iteratively subjecting it to analysis and validation can be highly informative. When a minimal model fails to fit experimental data or reproduce expected behaviours, the resulting discrepancy indicates that a key mechanism is missing. Through successive cycles of refining assumptions and hypotheses, reconstructing the model, analysing its behaviour, and comparing predictions with data, it becomes possible to identify which biological processes are essential for capturing the system’s dynamics. In this sense, encountering the “wrong” model at the outset can be advantageous, as it helps reveal the core components that drive the behaviour of the system.

Box 1 A practical workflow for productive collaboration between experimentalists and theoreticians Effective integration of experiments and modelling arises when both are developed together rather than sequentially. This requires continuous dialogue between experimentalists and modellers throughout the research process. The following principles can guide productive collaboration.

**Framing the problem**

- What biological phenomenon requires explanation?
- What is the central biological question?
- What observations must the model account for?
- Which hypotheses or mechanisms provide plausible starting points?

**Defining model structure**

- What is the minimal model required to address the question?
- Which biological processes must be represented explicitly?
- What simplifying assumptions and levels of abstraction are acceptable?
- How should uncertainty and biological variability be incorporated?

**Optimising experimental design**

- Which perturbations or experimental conditions best discriminate competing mechanisms?
- What temporal and spatial resolution (sampling frequency and duration) is needed?
- What are the major sources of experimental variability or noise?

**Aligning data and model**

- Do the available data sufficiently constrain the model?
- Are there more parameters than informative data points?
- Where are the largest sources of uncertainty?
- What additional measurements would most improve model discrimination and reduce uncertainty?

**Interpreting outcomes and refining the system understanding**

- What conclusions follow from data–model integration under the model’s assumptions?

- How do these conclusions align with existing biological knowledge?
- What aspects of the results remain uncertain or model-dependent?
- Which model predictions can be tested experimentally?

## Conclusion

The application of mathematical modelling frameworks is no longer confined to mathematically oriented disciplines; rather, it has become a necessary toolset for the broader biological community seeking to derive mechanistic insight from complex data. Moreover, the continued convergence of mechanistic modelling with data-driven methods based on machine-learning techniques and artificial intelligence offers a promising direction for uncovering hidden structure in complex datasets, optimising model discovery, and enhancing predictive power. Integrating spatially resolved omics, live-cell imaging, and dynamic perturbation experiments into these frameworks will be key to advancing mechanistic understanding. Ultimately, progress in this field will depend on iterative feedback between experimental, computational and theoretical efforts, fostering models that not only recapitulate observed phenomena, but also generate testable hypotheses and guide therapeutic design.

## Data Availability

No datasets were generated or analysed during the current study.
